# Circumscribed Traumatic Aneurism of a Tibial Artery

**Published:** 1891-03

**Authors:** Howard J. Williams


### CIRCUMSCRIBED TRAUMATIC ANEURISM OF A TIBIAL ARTERY.*

By HOWARD J. WILLIAMS, A. M., M. D.

### LIGATION OF THE FEMORAL ARTERY—RECOVERY.

I wish to present the following case of aneurism as the report of the Section on Surgery of the Macon Medical Association:

J. W. W., white, male, age thirty years, brakeman, while at work in the Central Railroad yards, on the night of November 30, 1889, received a gunshot wound in the calf of the left leg. At the time he was wounded he was standing about eight feet from two men who were fighting, so that his back and right side were exposed to their fire. The ball (a No. 38) entered the calf of the leg on a line corresponding to the junction of the upper and middle thirds of the leg, an inch and a half from the inner edge of the tibia, and making its exit at a point three-fourths of an inch from the anterior edge of the tibia, about an inch and a half below the above line. From the position of the wounds, I should judge that the ball passed between the tibia and fibula from behind forwards and from above downwards.

Dr. C. H. Hall attended him for the primary injury, and says there was very little hemorrhage, the wounds healing nicely and nothing unusual was noticed about the case.

The patient says, two weeks after the injury and after the wounds were united and Dr. Hall had discharged him, he noticed a slight swelling on the inside of the calf just below the wound of entrance. This swelling was soft, but was unattended by pain, throbbing, discoloration or heat. Its growth was rapid, soon reaching the size of half an orange. It then lost its softness, becoming more consistent or doughy.

*Read before Macon Medical Association, October, 1890.

About one month after the injury there appeared on the outside of the calf, just above the wound of exit and near the edge of the tibia, a slight pulsating spot, which grew in force and extent and became painful ; but it was not discolored, nor was it accompanied by heat. The pain increased in severity and extent, and was soon accompanied by boring pains in the tibia, ankle and foot. He became soon so disabled that he could only walk with difficulty, even with crutches.

I saw the patient first on January 24, 1890, nearly two months after he was wounded, and through the kindness of Dr. Hall took charge of the case.

At that time the swelling on the inside of the leg was about the size of half an orange, semi-soft and painless ; did not pulsate, and, applying the stethoscope, no sounds could be heard. The pulsating swelling on the outside of the leg was as large as an English walnut; pulsated synchronously with the heart's action, and a bruit could be distinctly heard. Pressure on the femoral artery obliterated the pulsation and bruit. There was no œdema of the foot, indicating no interference with the return circulation. No pulsation could be felt in the posterior tibial artery in the sulcus back of the internal maleolus. The circulation in this artery was therefore shut off below the internal or semi-solid mass. Pulsations in the anterior tibial and dorsalis pedis arteries could easily be felt.

The wound of exit and the pulsating tumor were under an extensive scar left by a sloughing wound following a dog bite received several years before. This scar gave the parts a glazed appearance, the cicatricial tissues being extremely thin ; and as the pulsation could be readily felt, it looked as though the aneurism was very near the surface and rupture was possible at any moment. The possibility of such an accident was favored by the patient's low general condition from prolonged suffering and anxiety. The necessity of immediate treatment was therefore indicated.

To give the patient all the chances of recovery without resorting to operative interference, I decided, after consultation with Dr. Hall, to employ pressure. I therefore bandaged the limb from the toes to above the knee with an ordinary muslin bandage and applied a tourniquet to the popliteal artery. The bandage was worn moderately tight continuously, while the tourniquet, tightened sufficiently to stop the pulsation and bruit, was worn for intervals of one to two hours, then relaxed for intervals of one-half to one hour. The patient was kept in bed with the limb in an elevated position. His diet was nutritious, but as dry a diet as it was possible for him to submit to. No medicines, such as iodide of potassium or aconite, were given him, as his general condition was too feeble to permit of further depression.

This treatment was borne persistently by the patient for several weeks with but little benefit. On the 24th of March, however, the pulsations and bruit suddenly ceased, and for three days thereafter they could not be detected. They then as suddenly returned, notwithstanding the fact that the treatment was still continuously pursued, and my hopes that the treatment was successful were sadly broken.

Pressure by the application of Esmarch's bandage suggested itself to my mind, but was objectionable, since gangrene of the parts and hemorrhage might possibly follow, owing to the thinness and low vitality of the cicatricial tissues covering the aneurismal sac. Such an accident might follow immediately on the application of the pressure or during a subsequent treatment by ligation, as has been reported in other cases.

I next thought of trying extreme flexion of the leg on the thigh, but, after eight hours of this method, continuously applied, I abandoned it. This treatment was extremely painful and tedious, the patient saying that he suffered more during that eight hours than during the entire several weeks of the first treatment. Nor did the flexion in any way moderate the pulsations, as the aneurism was too low down to be affected by the position.

After further consultation with Dr. Hall, I decided to ligate the femoral artery at Hunter's canal. Dr. McHatton was requested to see the case with me, as I wished his assistance in the operation. He agreed with me that ligation of the femoral artery would give the best chances of recovery.

March 16, 1890, assisted by Dr. McHatton and Dr. Derry, the latter administering ether, I ligated the femoral artery at Hunter's canal. Dr. Hall was unable to be present at the operation, owing to sickness.

Every antiseptic precaution was employed. The patient was required on the morning of the operation to take a thorough bath, shave the hair from the thigh and put on clean clothing. Clean bedding, clean towels and thoroughly boiled water were provided. The instruments to be used were placed for five minutes in hot water; our hands were thoroughly washed, first with soap and hot water; our nails trimmed and cleansed and then bathed with bichloride solution. Bichloride solutions for the sponges and douche were prepared. After the patient was placed on the operating table the tourniquet was applied loosely to the femoral in Scarpa's triangle, and he was etherized; the thigh was again washed with soap and water, then with ether, and lastly with bichloride solution (1 to 1,000).

The leg being adducted and flexed upon the thigh so as to bring the inside of the thigh upwards, the course of the femoral artery was outlined. An incision, beginning eight inches above the knee and extending three inches down the thigh, was made through the skin and superficial fascia. The deep fascia was next divided to the same extent on a grooved director, disclosing the fibers of the sartorius muscle. This muscle was pushed inwards, and I felt the pulsations of the artery at the bottom of the wound. The internal saphenous nerve, resting on the artery at this point, was pushed aside; a small opening in the sheath of the artery was made, and an aneurism needle bearing a silk ligature was passed around the artery, hugging the vessel closely to avoid the femoral vein which is here behind the femoral artery. Assuring ourselves that we had the vessel and that no vein or nerve was included, the ligature was drawn up and tied, leaving the ends of the ligature out of the wound. All pulsation in the aneurism was stopped by the application of the ligature. The wound was flushed with bichloride solution and closed with several silk sutures. The surface of the wound was freely dusted with iodoform; several layers of iodoform gauze, 8x8 inches square, covered the parts; next a layer of absorbent cotton was placed around the thigh, over which was placed a broad strip of rubber protective, and, finally, this dressing was kept in position by several turns of a roller bandage. The foot and entire limb was then enveloped in cotton to preserve the warmth of the extremity, and after the patient was placed in bed, several bottles of hot water were placed around the leg. The patient soon recovered consciousness and rested well.

At 6 P. M. the temperature was 99, pulse 100. Sensation of the extremity was good and the foot felt warm. The next two days (March 17th and 18th) the temperature and pulse remained normal; the color and sensations of the limb were good. The patient, however, complained occasionally of feeling hot flushes pass down the limb from the incision to the toes. The toes looked a little shrunken and dry, but not discolored.

March 19th: Temperature 100.4, pulse 114. Considerable mental depression and dread; complained of pain in the wound on the thigh, occasional hot flushes in the limb; color of limb good; shrunken appearance of toes gone. Feeble pulsations could be felt in the dorsalis pedis artery.

March 20th: Temperature 99.5, pulse, 100. Doing well. At noon was sent for, as it was thought that hemorrhage from the incision had occurred. I found a small bloody stain on the bandage and bed clothes, and on removing the dressings a large blistered surface 8x8 inches was found under the iodoform gauze. This blister had ruptured and, oozing from under the dressings, had given rise to the bloody stains. The incision in the thigh, which occupied the centre of the blistered surface and an area around it of an inch and a half, was not blistered, but was healthy, and primary union was taking place nicely. This wound and the healthy skin around it had been covered by pure powdered iodoform and luckily escaped the irritation of the gauze which had caused the blistering. The blister soon healed under dry iodoform freely dusted on it.

March 23d: Pulse and temperature normal. Removed the sutures. Primary union had taken place except where the ligature hung out of the wound. The solid tumor on the inside of the calf of the leg was rapidly disappearing. No pulsations could be felt in the aneurism. The pulsations of the dorsalis pedis artery could be distinctly felt.

March 26th: Feeble pulsations of the posterior tibial could be felt in the sulcus back of the internal maleolus.

April 7th: The ligature on the femoral artery came away. In a few days the wound left by the ligature had healed by granulation and the patient was out on crutches.

There has been no return of pulsation at the seat of the aneurism, and the patient is now well and has been at work, without the least inconvenience, for six months.

